# Anti-obesity effects of dichloromethane leaf extract of *Gnidia glauca* in high fat diet-induced obese rats

**DOI:** 10.1016/j.heliyon.2019.e02800

**Published:** 2019-11-21

**Authors:** Wycliffe Makori Arika, Cromwell Mwiti Kibiti, Joan Murugi Njagi, Mathew Piero Ngugi

**Affiliations:** a Department of Biochemistry, Microbiology and Biotechnology, School of Pure and Applied Sciences, Kenyatta University, P.O. Box 43844-00100, Nairobi, Kenya; b Department of Pure and Applied Sciences, Technical University of Mombasa, P.O. Box 90420 - 80100, Mombasa, Kenya; c Department of Environmental and Occupational Health, School of Environmental Sciences, Kenyatta University, P.O. Box 43844-00100, Nairobi, Kenya

**Keywords:** Toxicology, Chemistry, Food Science, Biological Sciences, Veterinary medicine, Health Sciences, Obesity, High-fat-diet (HFD), *Gnidia glauca*, Dichloromethane, Phytochemicals, And anthropometric indices

## Abstract

**Background:**

Obesity is a chronic metabolic disorder characterized by increased adipose tissue mass due to positive energy balance. Prescription of anti-obesity drugs can be useful adjuncts to diet and exercise for obese patients who have failed to achieve weight loss. However, these drugs are ineffective and are associated with adverse effects. In recent times, medicinal plants have drawn a sharp focus owing to their biocompatibility and effectiveness. Attempts to determine the therapeutic effects and identification of bio-active principles from herbal prescriptions have become the prime focus in the validation of their folkloric usage and in drug discovery programs. Therefore, the present study aimed to determine the anti-obesity effects of Dichloromethane leaf extract of *Gnidia glauca* in high-fat-diet-induced obese rats.

**Methods:**

Obesity was induced experimentally in white albino Wistar rats by feeding them with prepared high-fat-diet and water *ad libitum* for a period of 12 weeks. The *in-vivo* anti-obesity effects were determined by oral administration of *Gnidia glauca* at dosage levels of 200, 250 and 300 mg/kg body weight from the 6^th^ to 12^th^ week of study. Phytochemical analysis of *Gnidia glauca* was conducted using gas chromatography linked to mass spectrophotometer.

**Results:**

The results indicated that *Gnidia glauca* exhibited potent anti-obesity effects. It significantly reduced the body weight, organ weights, organo-somatic indices, anthropometric indices, the total fat content, adiposity index, atherogenic index as well as various lipid profiles. It also decreased the total feed intake. However, it significantly increased levels of high-density lipoproteins and rectal body temperature of rats. Quantitative phytochemical analysis also revealed the presence of various phytocompounds that have shown to be associated with anti-obesity effects.

**Conclusion:**

The anti-obesity effects of *Gnidia glauca* maybe attributed to the phytochemicals present. The present study, therefore, scientifically validates the traditional use of *Gnidia glauca* as a potential candidate for the synthesis of new effective anti-obesity supplement.

## 1 Introduction

Obesity is a chronic disease characterized by pathophysiological processes that result in increased adipose tissue mass due to positive energy balance [1]. Obesity involves an interaction of both genetic and environmental factors (such as behavioral, social, cultural, physiological) that can singly or synergistically contribute to its pathogenesis [2]. Fundamentally, obesity represents a phenotypic consequence of an energy imbalance between calories consumed and calories expended in favor of caloric intake [3]. These results may be due to increased consumption of energy-dense foods that are high in fat combined with decreased physical activity as a result of increasingly sedentary nature of many forms of work, transport, and urbanization [4].

The phenotypic manifestation of abdominal adiposity is linked with impairment of systemic metabolic homeostasis thereby resulting in reduced life expectancy and increased health complications [5]. Under obesogenic states, the hypertrophied and hyperplastic adipocytes result in an abnormal fat distribution that leads to weight gain equaling to or greater than 20% of the standard weight [6]. Altered regulatory signals of hunger and satiety, reduced thermogenesis and decreased resting metabolic rate over a prolonged period of time contributes to the propensity of an individual to become obese [7]. Hyperphagia, energy density, and post-ingestive effects of dietary fat are the main factors leading to dietary obesity [8].

Currently, obesity represents the most significant health promotion and disease prevention priorities worldwide due to increased risks of associated morbidity and mortality [1]. It presents modifiable risk factors for type II diabetes mellitus, coronary heart disease, hypertension, cognitive impairments, psychological disorders, musculoskeletal disorders (knee osteoarthritis) and certain cancers (endometrial, breast, and colon) [9, 10]. Although the medical consequences of obesity are of central concern to researchers and clinicians, obesity also appears to adversely affect an individual's capacity to live a full and active life [11]. It exacerbates an individual's psychological disorders such as poor self-esteem, depression, and poor employment prospects which substantially impacts a person's functional capacity and quality of life [11]. Once considered a high-income country burden, obesity is now dramatically on the rise in low- and middle-income countries, particularly in urban settings, and it's linked to more deaths worldwide than underweight [12].

The worldwide prevalence of obesity has more than doubled between 1980 and 2014. In 2014, more than 1.9 billion adults, 18 years and older, were overweight and at least 600 million of them were clinically obese [12]. Overall, about 13% of the world's adult population (11% of men and 15% of women) were obese in 2014 [12]. During the same year, 39% of adults aged 18 years and over (38% of men and 40% of women) were overweight [12]. In 2013, 42 million children under the age of 5 were overweight or obese and out of this, more than 30% were from developing countries [12]. Recent evidence indicates that overweight and obesity are increasing in Sub-Saharan Africa, including Kenya, at a rate of 5% per year on average [13]. On a global scale*,* obesity has been ranked as the fifth leading risk factor for global deaths and is projected to be the third leading cause of death by 2030 [12].

The procedures for qualitative and quantitative measurement of body adiposity ranges from simple and effective anthropometric methods (such as body mass index (BMI), waist circumference (WC), waist-to-hip ratio (WHR) and waist-to-height ratio (WHtR)) to magnetic resonance imaging and computerized technologies (such as Dual-energy X-ray Absorptiometry (DEXA) technique) [14]. These techniques are essential for diagnosis, risk assessment, and precise tracking of obese patients in terms of quantity and fat mass distribution [14]. Intra-abdominal fat thickness (visceral fat mass) has been shown to be a significant predictor of metabolic disturbances including; cardiovascular diseases, atherosclerosis, dyslipidemia, and hypertension [14]. Any significant change in the levels of lipids, atherogenic index, and body adiposity index predisposes individuals to the development of an atherosclerotic disease, endothelial dysfunction, as well as some cancers [14].

The mainstay of non-pharmacological treatment of obesity is diet, behavioral modification, and exercise program that individualize to the patient's lifestyle and physical needs [15]. However, prescription of anti-obesity drugs can be a useful adjunct for obese patients who have failed to achieve weight loss through diet and exercise program [16]. Despite the remarkable progress in the management of obesity by synthetic drugs, there has been a renewed interest in medicinal plants owing to their availability, affordability, and the easy biocompatibility, unlike the chemically synthesized drugs which are associated with adverse effects and numerous health hazards [16].

In African traditional medicine, *Gnidia glauca* has been therapeutically applied against many oxidative stress-related diseases such as sore throat, abdominal pain, wounds, burns, and snakebites [17]. It has also demonstrated significantly superior efficacy in the management of obesity. It has been a useful adjuvant and a key adjunct to dietary control in diabetic patients. The rationale for its utilization has rested largely on its long-term clinical experience. However, Ghosh *et al.* [18], have shown the plants’ strong biochemical basis in the management of diabetes mellitus through *in vitro* models. Further, using *in vitro* models, Ghosh *et al.* [19] have shown that the plant has antioxidative properties and that could explain its role in ameliorating oxidative stress-mediated diseases including obesity and its associated complications such as diabetes. However, there is no *in vivo* scientific information to validate the therapeutic effects of *Gnidia glauca* against obesity.

The development of *in vivo* preclinical models for obesity research such as polygenic models have provided insights into understanding mechanisms underpinning obesity development and obesity-associated syndromes [20]. Polygenic models are pathophysiologically relevant to human obesity following chronic exposures to a hypercaloric diet [20]. The behavioral phenotypes in obese animal models are characteristic of a positive energy balance in human population over many years [20]. Therefore, assessment of raw or isolated bio-active compounds in animal models of dietary obesity is essential in understanding their efficacy and safety profiles before validation in humans.

Numerous studies have been conducted in animal models of dietary obesity to establish the efficacy and safety profiles of herbal formulations. For instance, a study on the anti-obesity effects of *Ilex paraguariensis* (Yerba Mate) in C57BL/6J mice fed on a high-fat diet for 6 weeks revealed that Yerba Mate reduced food intake, decreased the differentiation of preadipocytes, reduced accumulation of lipids in adipocytes, increased energy expenditure and lowered body weight gain [21]. Similarly, administration of aqueous and ethanol leaf extracts of *Aegle marmelos* significantly decreased body weight, body mass index (BMI) and the waist-hip ratio of obese rats fed on high-fat diets (HFD) for 12 weeks [22]. The oral dose of 300 mg/kg/day of ethyl acetate and dichloromethane leaf extracts of *Murraya koenigii* significantly reduced the body weight gain, plasma levels of triglycerides (TGs) and total cholesterol (TC) in the high-fat diet (HFD) induced obese rats for 2 weeks [23]. Another study revealed that treatment of high-fat diet-induced obese rats with *Moringa oleifera* at doses of 200 mg and 400 mg/kg bodyweight for 7 weeks led to amelioration of obesity and associated complications with minimized adverse effects [24]. Consistent with such studies, application of the polygenic model of high methodological quality to determine the anti-obesity effects of *Gnidia glauca* and identification of active principles responsible for its activity is of prime focus in the validation of its folklore and drug discovery programs. The present study, therefore, aimed to determine the anti-obesity effects of Dichloromethanolic leaf extract of *Gnidia glauca* in high-fat diet-induced obese rats. The findings of this study will go a long way in providing ‘qualified leads’ in the synthesis of new and effective anti-obesity drug.

## 2 Methods

### 2.1 Collection of medicinal plant

Fresh leaves of *Gnidia glauca* were collected from their natural habitat at Makunguru Village, Nthawa Location, Siakago Division, Mbeere North Sub-County, Embu County, Kenya. The botanical identity of the plant was authenticated by a qualified taxonomist and a voucher specimen deposited at the National Museums of Kenya Herbarium for future reference. The specimen was assigned a voucher number as WAM-V1. Coordinates for the locations of collection points were taken using a hand-held GPS machine model type Garmin etrex H and recorded as shown in Table 1. The study was undertaken in the animal handling and experimental laboratory at the Department of Biochemistry, Microbiology and Biotechnology, Kenyatta University.

**Table 1:** Coordinates of Site of collection of the plant sample.

Plant species	UTM Eastings	UTM Northings	Latitude DMS	Longitude DMS
*G. glauca*	348,712.48	9,936,131.99	0^o^34′39.61”S	37^o^38′25.72″E

The coordinates of the location where the *Gnidia glauca* was sampled.

### 2.2 Processing and extraction of the plant material

Processing and extraction of the plant material was done according to the method described by Njogu *et al.* [25]. Fresh leaves of *G. glauca* were collected and shade-dried at room temperature for 21 days. The dried leaves were milled into fine powder by use of an electric mill. The powdered plant material was kept at room temperature away from direct sunlight in a dry airtight plastic container ready for extraction.

Five hundred grams of powdered *G. glauca* leaves were soaked in 1litre of dichloromethane for 48hrs. The solution was decanted and then filtered using muslin cloth into a different dry clean conical flask. The filtrate was concentrated under reduced pressure using a rotary evaporator at 40 °C to obtain a semi-solid residue [25]. The yield of the plant extract was determined and subsequently refrigerated at -20 °C until used for analysis.

### 2.3 Preparation of appropriate doses for bioassays

After a pilot study, the appropriate bioassay doses of DCM-leaf extracts of *G. glauca* for 5 animals per group were prepared by dissolving 0.23g in 2.5ml of 1% DMSO (200 mg/kg body weight), 0.29g in 2.5ml of 1% DMSO (250 mg/kg body weight), and 0.35g in 2.5ml of 1% DMSO (300 mg/kg body weight). Similarly, the dose of the reference drug, Orlistat, was prepared by dissolving 0.035g in 2.5ml of 1% DMSO (30 mg/kg body weight). The 1% DMSO was prepared by mixing 9ml of PBS with 1ml of 10% DMSO solution. In the entire dosing period, each experimental animal received a daily single-dose oral administration of 0.5ml of treatment at 0800hr. The choice for oral route of drug administration is based on the fact that it mimics the route of administration commonly used in the administration of *G. glauca* by herbalists to obese patients. All the treatment solutions were stored at -20 °C until used for bioassay.

### 2.4 Experimental animals

Thirty female white albino Wistar rats of about eight to ten weeks and weighing (mean ± SD) 120 ± 10g were ordered from Kenya Medical Research Institute (KEMRI) upon approval by the Ethics Committee for the Care and Use of Laboratory Animals of Kenyatta University, Kenya. Before initiation of the experiment, the rats were housed in groups of five in standard polypropylene cages maintained under controlled room temperature (23 ± 2 °C), bench level lighting of 360 lux and humidity (55 ± 5%) with 12hrs light and 12hrs dark cycle for one week for acclimatization [25, 26]. The lights were turned on at 0700 and off at 1900hrs. During this period, the rats were fed on standard laboratory diet, in the form of rodent pellets from Unga Feeds Limited, Nairobi, Kenya and water *ad libitum*. Environmental enrichment included paper wool nesting material and wood-shaving sawdust beddings. The rats were monitored thrice every day for health status. When no adverse events were indicated, the animals were weighed again before initiation of the experiment. The development of experimental protocols and procedures were performed under the guidance of the Veterinarian who is a member of the Veterinary Service Center (VSC) of Kenyatta University. All procedures were carried out in accordance with the Public Health Service (PHS) Policy on Humane Care and Use of Laboratory Animals as well as other local regulations laid down by the Institutional Animal Care and Use Committee (IACUC) (Section 8.3.2), Kenya veterinary Board and National Commission for Science, Technology and Innovation (NACOSTI).

### 2.5 Induction of obesity

Obesity was induced by feeding the experimental animals with a prepared High Fat Diet (HFD) and water *ad libitum* for 12 weeks. The composition of the experimental diet (g/kg diet) was done according to the formula described by Srinivasan [27] with some modifications as shown in Table 2. Rats with Lee obesity index value of 310 (equivalent to BMI in humans) and above were considered obese [28], and used in the study.

**Table 2:** Composition of high fat diet.

Ingredients	Diet (g/kg)
Powdered normal pellet diet	375
Lard	290
Casein	265
Corn oil	10
Cholesterol	10
Vitamin and mineral mix	60
DI Methionine	03
Yeast Powder	01
Sodium Chloride	01

The ingredients and their respective quantities of the prepared high fat diet.

### 2.6 Experimental design

In this study, a total of 30 rats were randomly grouped into six different groups of five rats each.

The G*Power software (2007) was used to calculate the sample size in each experimental group with parameters defined. Group I (normal control) consisted of normal rats maintained on standard chow diet for 12 weeks. This group of rats was administered with 1% DMSO. Group II (negative control) comprised of rats maintained on high fat diet for 12 weeks to induce obesity. This group was also administered with 1% DMSO as vehicle. Group III (positive control) consisted of HFD-induced obese rats treated with the reference drug, Orlistat, from the 6^th^ to 12^th^ week. Group IV-VI (experimental groups) comprised of HFD-induced obese rats that were administered with the DCM leaf extract of *G. glauca* at different doses of 200, 250 and 300 mg/kg body weight from the 6^th^ to 12^th^ week. During the entire dosing period, all the treated rats were maintained on a high fat diet. All the experimental rats received water *ad libitum* throughout the study period.

During the experimental period the body weight of each rat was assessed in grams after every seven days using a digital Mettler PJ 3000 weighing balance. The anthropometric and morphological measures were determined once every week. The obesity index was defined by Lee index. The Lee index was calculated according to the formula described by Lee [28].

(1) $$\text{Lee index (\%)}=\frac{\sqrt[{3}]{\mathrm{Body}\;\mathrm{Weight}\;(g)}}{\mathrm{Nose}-\mathrm{to}-\mathrm{Anus}\;\mathrm{Length}\;\left(\mathrm{cm}\right)}\times 1000$$

Obesity was defined by a Lee index of greater than 310 [28]. Following exposure to HFD (except for normal control group) for 6 weeks, all the rats in the negative control, positive control and extract-administered experimental groups attained the target diagnostic value of obesity, indicating the end to the obesity induction phase. The Naso–Anal Length (NAL) (cm) of rats was measured by a non-extensible thread and readings taken using a ruler with an accuracy of 0.1 cm.

The abdominal circumference (AC) was assessed on the largest zone of the rat abdomen in front of the hindlegs using a non-extensible thread. During the entire period, the rats were placed in ventral position. The readings of both abdominal and thoracic circumferences were taken using a ruler with an accuracy of 0.1 cm as described by Novelli *et al.* [29].

Blood sampling for determination of weekly fasting blood glucose levels was carried out using the method described by Njogu *et al.* [25]. Lateral caudal vein tail bleeding was conducted with the subjects having been fasted for 8 h. The tail was sterilized with 70% alcohol and then nipped using a 24G/26G needle. Blood samples were obtained once in every 7 days. Fasting blood glucose was measured each time with a glucose analyzer model (Hypoguard, Woodbridge, England).

Rat body temperature was measured on the 84^th^ day by Rectal probe thermometry (inserting a thermometer into rectal cavity of a rat) [30]. Rectal temperatures were recorded at intervals of 30 min for two and half hours after feeding rats with a high fat diet and administration of the treatments.

To minimize pain, suffering and harm to the experimental animals, Carprofen, a non-steroidal anti-inflammatory drug was used as an analgesic drug. It was administered subcutaneously at a dosage level of 5 mg/kg body weight per day. Besides, general anesthesia was induced by intraperitoneal administration of a solution of ketamine (80 mg/kg body weight) and xylazine (5 mg/kg body weight).

On the day of sacrifice, all the animals were euthanized using an overdose of isoflurane in a glass vacuum desiccator following an overnight fast. The blood was drawn from the heart of each sacrificed rat through cardiac puncture (laterally or ventrally) using a 5 ml syringe with a 23G1 needle. The blood samples were collected into a carefully labelled vacutainer. A drop of blood from this sample was used to determine fasting blood glucose level using a glucose analyzer model (Hypoguard, Woodbridge, England). The blood was allowed to stand for 3 h to ensure complete clotting. The clotted blood was centrifuged at 3000 rpm for 10 min and the resulting supernatant stored at -20 °C until required for analysis of lipid profiles.

### 2.7 Determination of organ weights and relative organ to body weight (organo-somatic index)

The liver, kidneys, spleen, heart, lungs and brain were carefully dissected out and weighed using a digital Mettler PJ 3000 weighing balance. These organs were then stored in 10% neutral buffered formalin. The organ somatic index (relative organ to body weight) was calculated using the formulae described by Vani *et al.* [31].

(2) $$\text{Relative ​organ ​to ​body ​weight ​(\%)}=\frac{\text{Actual ​weight ​of ​the ​organ ​}\left(\text{g}\right)}{\text{Body ​weight ​of ​an ​individual ​rat ​on ​day ​of ​sacrifice ​}\left(\text{g}\right)}\times 100$$

### 2.8 Determination of adipose depots

A midline laparotomy was performed following the sagittal plane, the intestines were removed, and retroperitoneum was exposed. The parametrial fat pad was cut at the midpoint of the base of the uterus and trimmed away along the length of the horns of both left and right ovaries. The retroperitoneal fat pad was excised as a triangular section extending laterally from the lower pole of the kidneys. While the kidneys were pulled toward the midline, the perirenal fat pad, that is seen embedded with adrenal glands, was dissected out just above the kidneys. Mesenteric fat pad found along the small intestines was dissected (by pulling gently) from the duodenum till the end of the colon. Placing the animal on its side, the flap of skin just below the ribcage was carefully peeled back rostrally exposing the inguinal subcutaneous fat pad which was dissected away from the underlying muscle. Finally, the BAT was dissected away from the interscapular depot in the neck region of the animal.

The four dissected intra-abdominal white fat pads (visceral WAT depots) (the retroperitoneal, perirenal, parametrial and mesenteric) as well as the one excised subcutaneous white fat pad (inguinal) and BAT interscapular depot were weighed using Mettler PJ 3000 weighing balance. The weights of these tissues were combined to form the *ex-vivo* Fat Mass.

As a measure of adiposity, the Body Adiposity Index (BAI) was calculated by using the formula described by Singh *et al.* [32].

(3) $$\text{BAI ​(\%)}=\frac{\sum \text{Mesenteric}+\text{Retroperitoneal}+\text{Parameterial}+\text{Perirenal}+\text{Subcutaneous}+\text{BAT}\left(\text{g}\right)}{\text{Final ​Body ​Weight ​}\left(\text{g}\right)}\times 100$$

### 2.9 Determination of lipid profiles

The separated sera were used for estimation of lipid profile such as the total cholesterol (TC), LDL-cholesterol, HDL-cholesterol and triglycerides (TAG) levels. These parameters were determined using Olympus 640 chemistry auto analyzer based on the standard operating procedures (SOPs) written and maintained in the Department of Laboratory Medicine, Kenyatta National Hospital.

The levels of serum Very Low-Density Lipoprotein (VLDL) were calculated in accordance with a formula as described by Friedewald and Fredrickson [33].

(4) $$\text{VLDL ​}=\text{ ​}\frac{\text{Triglycerides ​}\left(\text{TG}\right)}{5}$$

Atherogenic index of plasma (AIP) was calculated using the formula as described by Nwagha [34].

(5) $$\text{Atherogenic ​index ​(AIP)}=\text{log ​}\left(\frac{\text{Triglycerides}}{\text{HDL}-\text{C}}\right)$$

### 2.10 Determination of feed intake

All the food intake was measured daily for the period of 12 weeks at the same time on per cage basis and the average food consumed calculated. Each animal was provided with each food component daily and feed intake determined by the food remnants method, with the remnant feed being weighed before and after diet consumption. A digital Mettler PJ 3000 weighing balance was used for taking measurements throughout the study period.

### 2.11 Gas chromatography – mass spectrometry (GC-MS) analysis

Analysis of sample was carried out using GC-MS (7890/5975 Agilent Technologies, Inc., Beijing, China) consisting of a gas-chromatography interfaced to a mass spectrometer instrument. The GC-MS was equipped with a HP-5 MS (5% phenyl methyl siloxane) low bleed capillary column of 30m length, 0.25mm diameter and 0.25μm film thickness. For GC-MS detection, an electron ionization system with ionization energy of 70Ev was used. The carrier gas used was helium (99.99%) at a constant flow rate of 1.25 ml/min in split mode. The injector and mass transfer line temperature were set at 250 °C and 200 °C respectively, and an injection volume of 1 μl was employed. The oven temperature was programmed from 35 °C for 5 min, with an increase of 10 °C/min to 280 °C for 10.5 min, then 50 °C/min to 285 °C for 29.9 min with a run time of 70 min. The mass spectrometry operating parameters were as follows: ionization energy, 70eV; ion source temperature, 230 °C; solvent cut time, 3.3 min; relative detector gain mode; scan speed, 1666 μ/sec; scan range of 40–550 m/z and the interface temperature of 250 °C.

### 2.12 Data management and statistical analysis

The data on the anti-obesity effect of DCM leaf extract of *G. glauca* in HFD-induced obese rats was entered in the Microsoft® Excel spreadsheet, where it was organized and then exported to statistical software Minitab for analysis. The data was found to conform to the assumptions of parametric data. One-way ANOVA was used to test the significance among the normal control group rats, negative control group rats, Orlistat-treated group of rats and extract-treated group of rats. The data was further subjected to Tukey's post hoc for pairwise comparison and separation of means. The results were expressed as the Mean ± Standard Deviation (SD) and presented in tables and graphs. All differences were considered statistically significant if *p* ≤ 0.01. The Minitab software (Version 17.1, NC, USA) was used to perform all the statistical analyses. Phytocompound identities were proposed based on their general fragmentation pattern and using reference spectra published by library– mass spectral databases [National Institute of Standards and Technology (NIST) library version (2005), software, Turbomas 5.2].

## 3 Results

### 3.1 Effects of DCM leaf extract of *Gnidia glauca* on body weights of HFD-Induced obese rats

**Fig. 1:**
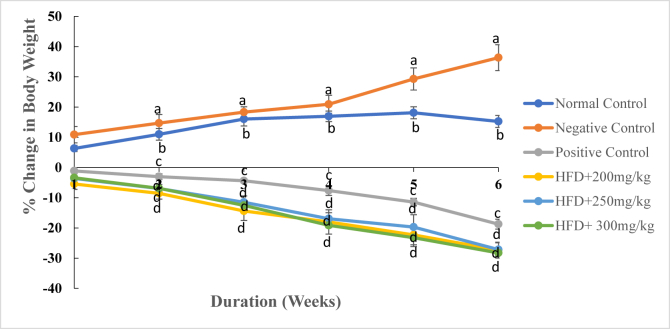
The mean percentage change in body weights of experimental rats treated with the DCM leaf extract of *Gnidia glauca* for 6 weeks. Each point on the curve represents the replicate measurement n = 5. The mean percentage changes in body weights of animals are expressed as Mean ± S.D per week. The weekly percentage changes in body weights of rats among the studied groups with a similar lower-case letter (such as ‘**d**’) are not significantly different from each other (*p* > 0.01). However, the weekly percentage changes in body weights of rats with different lower-case letters (such as **a**, **b**, **c** & **d**) indicates statistical difference in means among the studied groups (*p* ≤ 0.01). Inferential statistics were performed using One way-ANOVA to test for statistical differences among studied groups. Tukey's post hoc test was then done for pairwise separation and comparison of means.

**Fig. 2:**
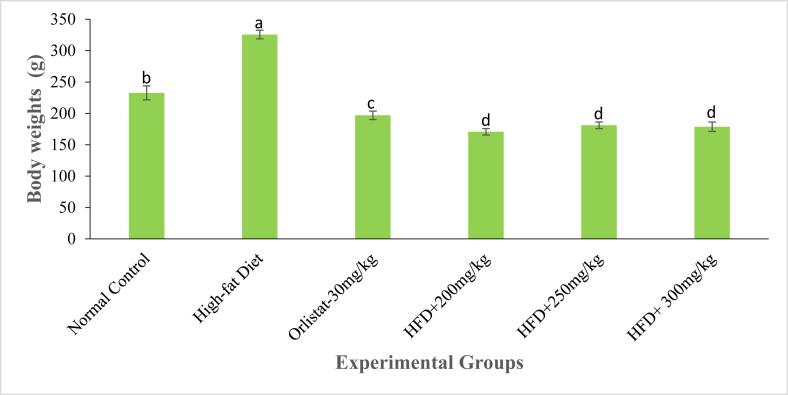
The body weights (g) of experimental animals on the 6^th^ week of treatment with the DCM leaf extract of *Gnidia glauca*. Each bar graph represents the mean replicate measurement n = 5 expressed as Mean ± S.D. The bar graphs with a similar lower-case letter (such as ‘**d**’) among experimental groups are not significantly different from each other (*p* > 0.01). The bar graphs with different lower-case letters (such as **a**, **b**, **c** & **d**) are statistically different from each other (*p* ≤ 0.01). Inferential statistics were performed using One way-ANOVA to test for statistical differences among studied groups. Tukey's post hoc test was then done for pairwise separation and comparison of means.

As Fig. 1 and Table 3 shows, during the first week of study, the rate of weight gain in the normal control rats significantly differed from the gain in body weight in the second week (*p* ≤ 0.01). However, the rate of weight gain in the same group of rats did not differ significantly in the 3^rd^, 4^th^, 5^th^ and 6^th^ week of the study period (*p* ≤ 0.01; Fig. 1; Table 3). In the negative control group, there was a marked increase in body weight throughout the study period. Indeed, the rate of weight gain ranged from 10.87 ± 2.71 % in the 1^st^ week to 36.34 ± 4.27 % in the 6^th^ week (Fig. 1; Table 3). On the other hand, treatment of rats with the reference drug, Orlistat and the three extract doses caused a decrease in body weights from the 1^st^ to the 6^th^ week of the study period (Fig. 1; Table 3). The rat models administered with the DCM leaf extract of *G. glauca* at a dose of 200 mg/kg body weight showed negative changes in body weight from -5.45 ± 1.79 % in the 1^st^ week to -27.32 ± 2.63 % in the 6^th^ week of study (Fig. 1; Table 3). On the last week of study, the body weight of rats in the negative control group was significantly higher than those of extract-treated rats, Orlistat-treated rats and rats in the normal control group (*p* ≤ 0.01; Fig. 2; Table 3). Besides, rats treated with the three extract doses recorded the lowest body weights relative to other experimental groups (Fig. 2; Table 3).

**Table 3:** Effect of oral administration of DCM leaf extract of *Gnidia glauca* for 6 weeks on body weight of HFD-induced obese laboratory rats.

TREAT-MENTS (mg/kg)	Weekly body weights of rats (g)						
	Day 1	Day 7	Day 14	Day 21	Day 28	Day 35	Day 42
Control	191.3 ± 6.9^b^	212.1 ± 10.1^c^	219.4 ± 10.8^bc^	223.9 ± 11.6^b^	227.9 ± 11.8^b^	230.1 ± 11.4^b^	232.6 ± 11.2^b^
High-fat Diet	233.9 ± 4.4^a^	259.4 ± 4.9^a^	265.1 ± 4.7^a^	277.9 ± 1.9^a^	288.8 ± 3.7^a^	308.8 ± 3.4^a^	325.6 ± 6.9^a^
Orlistat	242.5 ± 10.1^a^	239.7 ± 10.8^ab^	235.2 ± 10.4^b^	231.8 ± 8.4^b^	223.9 ± 6.3^b^	214.7 ± 7.7^bc^	196.9 ± 6.7^c^
200 mg/kg	235.1 ± 11.4^a^	222.3 ± 12.5^bc^	214.9 ± 10.9^c^	201.2 ± 11.9^c^	192.7 ± 12.1^c^	182.7 ± 13.5^d^	170.6 ± 5.1^d^
250 mg/kg	248.6 ± 5.8^a^	239.6 ± 5.9^ab^	231.3 ± 4.9^bc^	220.0 ± 3.8^b^	206.4 ± 6.7^c^	199.5 ± 8.3^cd^	181.1 ± 5.3^d^
300 mg/kg	249.1 ± 9.4^a^	240.7 ± 9.0^a^	231.9 ± 7.6^b^	217.8 ± 5.3^b^	201.5 ± 7.7^c^	191.2 ± 4.9^d^	178.7 ± 7.5^d^

Results are expressed as Means ± SD for five animals per group. The means of body weight of experimental animals within respective columns followed by superscript of a similar lower-case letter (such as [**a** & **a**b], [**b**, a**b** & **b**c], [**c**, b**c** & **c**d] or [**d**, c**d**]) are not significantly different from each other (*p* > 0.01). However, in each column, means followed by superscripts of different lower-case letter (such as **a**, **b**, **c** & **d**) are significantly different from each other (*p* ≤ 0.01). Inferential statistics were performed using One way-ANOVA to test for statistical differences among studied groups. Tukey's post hoc test was then done for pairwise separation and comparison of means.

### 3.2 Effect of DCM leaf extract of *Gnidia glauca* on anthropometric measures in HFD-Induced obese rats

**Fig. 3:**
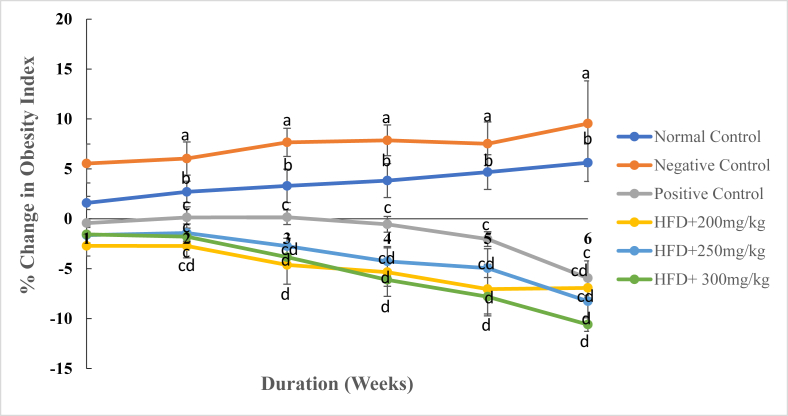
The mean percentage change in obesity index of experimental animals treated with the DCM leaf extract of *Gnidia glauca* for 6 weeks. Each point on the curve represents the mean of the replicate measurement n = 5 expressed as Mean ± S.D. The weekly percentage changes in obesity index among the studied groups with a similar lower-case letter (such as [**c**, **c**d] or [**d** & c**d**] are not significantly different from each other (*p* > 0.01). However, the weekly percentage changes in obesity index of rats with different lower-case letters (such as **a**, **b**, **c** & **d**) indicates statistical difference in means among the studied groups (*p* ≤ 0.01). Inferential statistics were performed using One way-ANOVA to test for statistical differences among studied groups. Tukey's post hoc test was then done for pairwise separation and comparison of means.

**Fig. 4:**
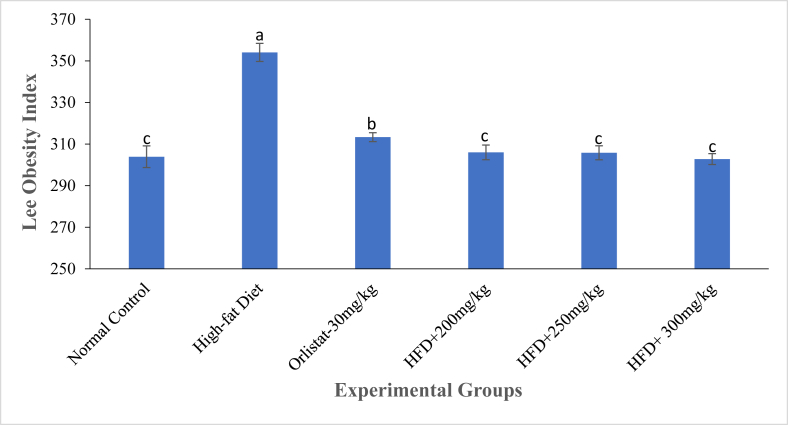
The Lee obesity index of experimental animals on the 6^th^ week of treatment with the DCM leaf extract of *Gnidia glauca*. Each bar graph represents the mean replicate measurement n = 5 expressed as Mean ± S.D. The bar graphs with a similar lower-case letter (such as ‘**c**’) among experimental groups are not significantly different from each other (*p* > 0.01). The bar graphs with different lower-case letters (such as **a**, **b** & **c**) are statistically different from each other (*p* ≤ 0.01). Inferential statistics were performed using One way-ANOVA to test for statistical differences among studied groups. Tukey's post hoc test was then done for pairwise separation and comparison of means.

**Fig. 5:**
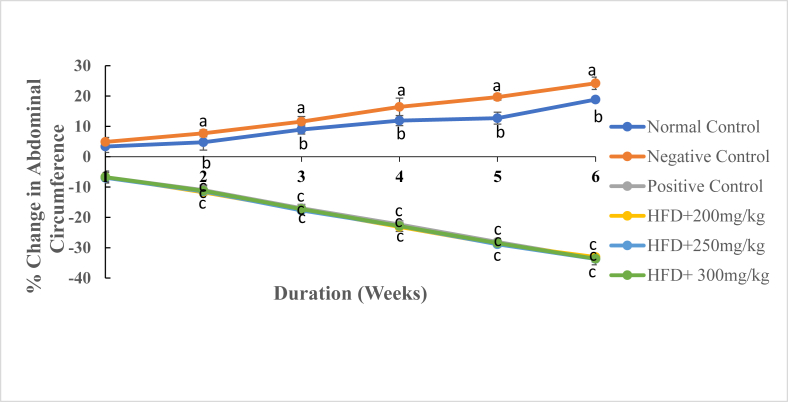
The mean percentage change in Abdominal Circumference (AC) of experimental animals treated with the DCM leaf extract of *Gnidia glauca* for 6 weeks. Each point on the curve represents the mean of the replicate measurement n = 5 expressed as Mean ± S.D. The weekly percentage changes in abdominal circumference of rats among the studied groups with a similar lower-case letter (such as ‘**c**’) are not significantly different from each other (*p* > 0.01). However, the weekly percentage changes in abdominal circumference of rats with different lower-case letters (such as **a**, **b** & **c**) indicates statistical difference in means among the studied groups (*p* ≤ 0.01). Inferential statistics were performed using One way-ANOVA to test for statistical differences among studied groups. Tukey's post hoc test was then done for pairwise separation and comparison of means.

**Fig. 6:**
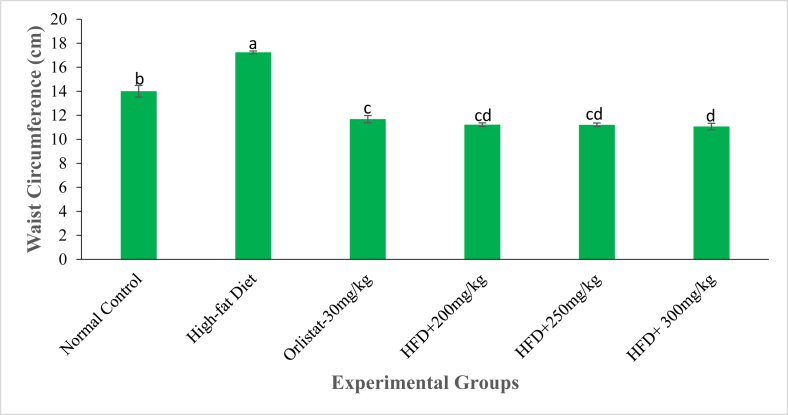
The abdominal circumference (cm) of experimental animals on the 6^th^ week of treatment with the DCM leaf extract of *Gnidia glauca*. Each bar graph represents the mean replicate measurement n = 5 expressed as Mean ± S.D. The bar graphs with a similar lower-case letter (such as [**c** & **c**d] or [**d** & c**d**) among experimental groups are not significantly different from each other (*p* > 0.01). The bar graphs with different lower-case letters (such as **a**, **b**, **c** & **d**) are statistically different from each other (*p* ≤ 0.01). Inferential statistics were performed using One way-ANOVA to test for statistical differences among studied groups. Tukey's post hoc test was then done for pairwise separation and comparison of means.

Generally, changes in anthropometric parameters were observed following treatment of HFD-induced obese rats with DCM leaf extract of *Gnidia glauca* (Figs. 3, 4, 5, and 6). The results showed an increase in obesity index in the normal and negative control group of rats from the 1^st^ to the 6^th^ week of treatments (Fig. 3). Conversely, treatment of rats with the reference drug, Orlistat, and the three doses of the plant extract caused a persistent decrease in the obesity index from the first to the last week of the study period (Fig. 3).

In the negative control group, obesity index increased from 5.54 ± 1.946 % in the 1^st^ week to 9.54 ± 4.28% in the 6^th^ week of study. The normal control group rats also showed an increase of the obesity index from 1.59 ± 0.66% in the 1^st^ week to 5.62 ± 1.88% in the 6^th^ week (Fig. 3). Administration of the extract at doses of 250 and 300 mg/kg body weight caused a negative change in obesity index from -1.63 ± 0.77% and -1.56 ± 0.29% in the 1^st^ week to -8.25 ± 1.50% and -10.59 ± 0.68% in the 6^th^ week of study respectively (Fig. 3). Similarly, rats treated with the reference drug, Orlistat, caused a reduction in obesity index from -0.43 ± 0.29% in the 1^st^ week to -5.94 ± 1.73% in the 6^th^ week of treatment (Fig. 3). Analysis of the Lee obesity index on the 6^th^ week of study revealed that the HFD-fed untreated obese rats exhibited an increased obesity index relative to other groups (Fig. 4). During this period, however, the extract-treated rats indicated relatively reduced Lee obesity index than Orlistat-treated rats and normal control group rats (Fig. 4).

It was observed that the abdominal circumference of rats in the normal control and negative control groups persistently increased in the entire study period (Fig. 5). Conversely, rats treated with the reference drug, Orlistat, and those treated with three extract doses indicated a decrease in abdominal circumference throughout the study period (Fig. 5). In the normal control group, there was a marked increase in abdominal circumference from 3.36 ± 1.95% in the 1^st^ week to 18.85 ± 0.92% in the 6^th^ week of study (Fig. 5). Similarly, in the negative control group, the abdominal circumference of rats increased from 4.88 ± 1.47% in the 1^st^ week to 24.22 ± 2.04% in the 6^th^ week of study (Fig. 5). The extract-treated groups of rats and Orlistat-treated rats showed significant reductions in abdominal circumference from the first to the last week of treatments (*p* ≤ 0.01; Fig. 5). On the 42^nd^ day, the abdominal circumference of extract-treated rats was significantly low as compared to those of rats in the negative control group (Fig. 6). Moreover, rats administered with the highest concentration of the extract (300 mg/kg) showed the lowest abdominal circumference in the same week of study (Fig. 6).

### 3.3 Effect of DCM leaf extract of *G. glauca* on organ weights and relative organ to body weights of HFD-Induced obese rats

The results showed that treatment with the DCM leaf extract of *G. glauca* caused changes in organ weights of HFD-induced obese rats (Table 4a). The weights of the liver, kidney, heart, lungs and brain were significantly higher in the negative control group rats than the normal control group rats, Orlistat-treated rats and extract-treated rats (*p* ≤ 0.01; Table 4a). On the contrary, the weight of the spleen was significantly low in the negative control group rats relative to that of rats in the normal control, positive control and extract-treated groups (*p* ≤ 0.01; Table 4a).

**Table 4a:** Effects of DCM leaf extract of *Gnidia glauca* on organ weights of HFD-Induced obese rats.

TREATMENT (mg/kgbw)	Organ weights (g)					
	Liver	Kidneys	Spleen	Heart	Lungs	Brain
Normal Control	10.3 ± 0.4^b^	2.0 ± 0.1^b^	1.1 ± 0.1^a^	1.0 ± 0.1^b^	2.9 ± 0.3^b^	2.1 ± 0.1^b^
Negative Control	23.1 ± 0.7^a^	5.6 ± 0.2^a^	0.2 ± 0.1^b^	3.8 ± 0.3^a^	9.1 ± 0.4^a^	4.6 ± 0.4^a^
Positive Control	6.2 ± 0.4^c^	1.6 ± 0.1^bc^	0.7 ± 0.5^a^	0.5 ± 0.1^bc^	2.2 ± 0.1^c^	1.6 ± 0.1^bc^
HFD+200 mg/kg	6.7 ± 0.2^c^	1.3 ± 0.1^bc^	0.7 ± 0.5^a^	0.5 ± 0.1^bd^	1.9 ± 0.1^cd^	1.5 ± 0.1^bc^
HFD+250 mg/kg	6.6 ± 0.3^c^	1.1 ± 0.1^c^	0.7 ± 0.3^a^	0.4 ± 0.1^de^	1.8 ± 0.1^d^	1.5 ± 0.1^bc^
HFD+ 300 mg/kg	6.1 ± 0.1^c^	1.1 ± 0.4^c^	0.6 ± 0.1^a^	0.3 ± 0.1^e^	1.5 ± 0.1^d^	1.4 ± 0.1^c^

Results are expressed as Means ± SD for five animals per group. The means of organ weights within respective columns followed by a superscript of similar lower-case letter (such as [**a**], [**b**, a**b, b**c & **b**d], [**c**, b**c** & **c**d], [**d**, b**d** & c**d**] or [**e** & d**e**]) are not significantly different from each other (*p* > 0.01). However, in each column, means followed by superscripts of different lower-case letter (such as **a**, **b**, **c, d** & **e**) are significantly different from each other (*p* ≤ 0.01). Inferential statistics were performed using One way-ANOVA to test for statistical differences among studied groups. Tukey's post hoc test was then done for pairwise separation and comparison of means.

The extract-treated rats indicated a significantly lower weight of the liver than the normal control group rats (*p* ≤ 0.01). However, the weight of the liver of extract-treated rats was not statistically significant from that of Orlistat-treated rats (*p* > 0.01; Table 4a). Similarly, there was no significant difference in the weights of the kidneys and the brain between extract-treated rats and Orlistat-treated rats (*p* > 0.01; Table 4a). However, the weights of the two organs were significantly higher in the normal control group rats than extract-treated group of rats (*p* ≤ 0.01). The weight of the heart of rats treated at extract dosages of 250 and 300 mg/kg body weight was statistically significant to that of rats treated with the reference drug, Orlistat (*p* ≤ 0.01; Table 4a).

There was a significant decrease in weight of lungs in extract-treated rats relative to the negative control group rats (*p* ≤ 0.01). The extract lowered the weight of lungs of rats in all the three extract doses than as observed in normal control group rats (*p* ≤ 0.01). However, the weights of lungs of rats administered with the three extract doses was not significantly different from that of Orlistat-treated rats (*p* > 0.01; Table 4a).

The DCM leaf extract of *G. glauca* also caused changes in relative-organ weights (organo-somatic index) of HFD-induced obese rats (Table 4b). The organo-somatic indices of liver, kidney, heart, lungs and brain of rats in the negative control group were significantly higher than those of rats administered with reference drug, Orlistat, and the three extract doses of *G. glauca* (*p* ≤ 0.01; Table 4b). However, the organo-somatic indices of spleen were significantly low in the negative control group rats relative to extract-treated and Orlistat-treated groups of rats (*p* ≤ 0.01; Table 4b). The effect of the extract was as effective as that of the reference drug, Orlistat, on the organo-somatic indices of the kidneys, spleen and brain (*p* > 0.01; Table 4b). Further, the extract-treated rats showed significantly lower weight of liver than normal control group rats and Orlistat-treated rats (*p* ≤ 0.01; Table 4b).

**Table 4b:** Effects of DCM Leaf Extract of *Gnidia glauca* on Relative-Organ Weights (somatic index) of HFD-Induced Obese Rats.

TREATMENT (mg/kgbw)	Relative Organ to Body Weights (Somatic Index) (%)					
	Liver	Kidney	Spleen	Heart	Lungs	Brain
Normal Control	4.5 ± 0.4^b^	0.9 ± 0.1^b^	0.5 ± 0.1^a^	0.4 ± 0.0^b^	1.2 ± 0.1^b^	0.9 ± 0.1^b^
Negative Control	7.1 ± 0.1^a^	1.7 ± 0.1^a^	0.1 ± 0.0^b^	1.2 ± 0.1^a^	2.8 ± 0.1^a^	1.4 ± 0.1^a^
Positive Control	3.2 ± 0.2^e^	0.8 ± 0.1^b^	0.4 ± 0.2^a^	0.3 ± 0.0^b^	1.1 ± 0.1^bc^	0.9 ± 0.1^bc^
HFD+200 mg/kg	3.9 ± 0.2^c^	0.8 ± 0.1^b^	0.4 ± 0.3^a^	0.3 ± 0.0^b^	1.1 ± 0.0^bc^	0.8 ± 0.1^bc^
HFD+250 mg/kg	3.7 ± 0.4^cd^	0.7 ± 0.1^b^	0.3 ± 0.1^ab^	0.2 ± 0.0^c^	1.0 ± 0.0^cd^	0.8 ± 0.0^bc^
HFD+ 300 mg/kg	3.4 ± 0.1^de^	0.7 ± 0.1^b^	0.2 ± 0.1^ab^	0.2 ± 0.0^c^	0.9 ± 0.1^d^	0.7 ± 0.1^c^

Results are expressed as Means ± SD for five animals per group. The means of organo-somatic indices within respective columns followed by a superscript of similar lower-case letter (such as [**a** & **a**b], [**b** & **b**c], [**c**, b**c** & **c**d], [**d** & c**d**] or [**e** & d**e**]) are not significantly different from each other (*p* > 0.01). However, in each column, means followed by superscripts of different lower-case letter (such as **a**, **b**, **c, d** & **e**) are significantly different from each other (*p* ≤ 0.01). Inferential statistics were performed using One way-ANOVA to test for statistical differences among studied groups. Tukey's post hoc test was then done for pairwise separation and comparison of means.

Findings also indicated that the organo-somatic index of hearts of rats treated with the extract dose of 200 mg/kg body weight were statistically similar to those of rats in the normal control group and Orlistat-treated group of rats (*p* > 0.01; Table 4b). However, the organo-somatic indices of the hearts at the extract dosages of 250 and 300 mg/kg body weight were significantly lower than those of rats in the normal control group and Orlistat-treated group of rats (*p* ≤ 0.01; Table 4b). Notably, the organo-somatic indices of lungs of extract-treated rats at the dose of 200 mg/kg body weight were statistically similar to those of the normal control group rats and Orlistat-treated rats (*p* > 0.01; Table 4b). The administration of extract doses of 250 and 300 mg/kg body weight significantly lowered the organo-somatic indices of lungs as compared to those of rats in the normal control group (*p* ≤ 0.01; Table 4b).

### 3.4 The effect of DCM leaf extract of *G. glauca* on total fat content in HFD-Induced obese laboratory rats

The weights of different fat pads such as mesenteric, retroperitoneal, parametrial, perirenal, subcutaneous and total fat content varied differently across the experimental groups (Table 5). The weight of mesenteric fat pad was significantly higher in HFD-induced obese untreated rats than all other experimental groups (*p* ≤ 0.01; Table 5). The weight of mesenteric fat pad was significantly lower in the extract-treated group of rats than that of rats treated with the reference drug, Orlistat (*p* ≤ 0.01; Table 5). However, there was no significant difference in weight of mesenteric fat pad of rats in the normal control and rats treated with the reference drug, Orlistat (*p* > 0.01; Table 5). Similarly, the weight of mesenteric fat pads in the group of rats treated with 200 mg/kg body weight extract dose and the reference drug, Orlistat were comparable (*p* > 0.01; Table 5). It was also observed that the weight of retroperitoneal fat pad was increased in the negative control group of rats relative to those of rats in the normal control, positive control and extract-treated groups (*p* ≤ 0.01; Table 5).

**Table 5:** Effects of DCM leaf extract of *Gnidia glauca* on total fat content in HFD-Induced obese rats.

TREATMENT (mg/kgbw)	Fat Pad Weight (g)						
	MESENTERIC	RETROPERITONEAL	PARAMETRIAL	PERIRENAL	SUBCUTANEOUS	BAT	TOTAL FAT
Normal Control	2.8 ± 0.3^b^	1.3 ± 0.1^b^	0.3 ± 0.1^b^	2.2 ± 0.3^b^	2.6 ± 0.1^d^	0.3 ± 0.1^b^	9.4 ± 0.3^bc^
Negative Control	5.9 ± 0.3^a^	4.1 ± 0.1^a^	0.4 ± 0.1^a^	4.1 ± 0.3^a^	6.7 ± 0.2^a^	1.2 ± 0.1^a^	22.5 ± 0.4^a^
Orlistat-30 mg/kg	2.6 ± 0.1^bc^	1.3 ± 0.1^b^	0.2 ± 0.1^c^	2.0 ± 0.2^b^	3.9 ± 0.3^b^	0.2 ± 0.0^c^	10.0 ± 0.4^b^
HFD+200 mg/kg	2.2 ± 0.2^cd^	1.4 ± 0.2^b^	0.2 ± 0.0^c^	1.9 ± 0.1^bc^	3.2 ± 0.4^c^	0.1 ± 0.1^c^	9.000.8^c^
HFD+250 mg/kg	2.0 ± 0.2^d^	1.2 ± 0.1^b^	0.1 ± 0.01^c^	1.5 ± 0.1^cd^	2.9 ± 0.2^cd^	0.1 ± 0.0^c^	7.8 ± 0.3^d^
HFD+ 300 mg/kg	1.9 ± 0.1^d^	1.3 ± 0.3^b^	0.1 ± 0.0^c^	1.4 ± 0.1^d^	2.7 ± 0.2^d^	0.1 ± 0.1^c^	7.4 ± 0.3^d^

Results are expressed as Means ± SD for five animals per group. The means of fat pad weights within respective columns followed by a superscript of similar lower-case letter (such as [**a**], [**b** & **b**c], [**c**, b**c** & **c**d] or [**d** & c**d**]) are not significantly different from each other (*p* > 0.01). However, in each column, means followed by superscripts of different lower-case letter (such as **a**, **b**, **c** & **d**) are significantly different from each other (*p* ≤ 0.01). Inferential statistics were performed using One way-ANOVA to test for statistical differences among studied groups. Tukey's post hoc test was then done for pairwise separation and comparison of means.

The parametrial fat pad weights were significantly higher in the negative control group rats than rats treated with the three extract doses (*p* ≤ 0.01; Table 5). Further, all extract treated groups of rats exhibited significantly lower weights of parametrial fat pad than the normal control group rats (*p* ≤ 0.0; Table 5). However, no significant difference in weight of parametrial fat pad was indicated between rats treated with the reference drug, Orlistat, and those treated with the three extract doses (*p* > 0.01; Table 5).

The weights of perirenal fat pads were observed to be significantly higher in the negative control rats than those of rats in the normal control, positive control and extract-treated groups (*p* ≤ 0.01; Table 5). Treatment of rats with the extract dose of 300 mg/kg body weight led to the lightest perirenal fat pad (Table 5). The activities of the reference drug, Orlistat, in reduction of weights of the perirenal fat pad were comparable to that of the extract at a dose of 200 mg/kg body weight (*p* > 0.01; Table 5).

The weight of subcutaneous fat pads in HFD-induced untreated obese rats was significantly high as compared to those of rats in the normal control, positive control and extract-treated groups (*p* ≤ 0.01; Table 5). Remarkably, all the concentrations of the plant extract indicated significantly reduced weights of the subcutaneous fat pad than those of rats treated with the reference drug, Orlistat (*p* ≤ 0.01; Table 5). The highest concentration (300 mg/kg body weight) of the extract showed the lightest weight of the subcutaneous fat pad (Table 5).

Treatment with DCM leaf extract of *G. glauca* significantly reduced the weight of brown adipose tissue (BAT) fat pads in HFD-induced obese rats relative to the negative control obese untreated rats (*p* ≤ 0.01; Table 5). As shown, the highest extract dose recorded the lowest weight of BAT (Table 5). However, no significant change in weight of BAT was observed between rats treated with the reference drug, Orlistat, and those treated with the three extract doses (*p* > 0.01; Table 5).

The results revealed that rats in the negative control group showed significantly higher weight of the total fats than those of rats in the normal control, positive control and extract-treated groups (*p* ≤ 0.01; Table 5). The weights of the total fats gradually reduced as the concentration of the plant extracts increased with the highest dose of 300 mg/kg body weight recording the lightest weight of total fats (Table 5).

### 3.5 Effect of DCM leaf extract of *Gnidia glauca* on lipid profiles in HFD-Induced obese rats

It was observed that administration of DCM leaf extract of *G. glauca* in HFD-induced obese rats altered the levels of serum lipid profiles and blood glucose (Table 6). The results showed that there was a significant increase in triglycerides (TG) in the negative control group rats relative to the normal control, negative control and the extract-treated groups rats (*p* ≤ 0.01; Table 6). Findings also indicated that the reduction of triglycerides in the positive control group were statistically similar to that of the extract-treated groups (*p* > 0.01). Treatment with the extract at dosage levels of 250 and 300 mg/kg body weight significantly reduced the triglycerides levels as compared to the normal control group (*p* ≤ 0.01; Table 6).

**Table 6:** Effect of oral administration of DCM leaf extract of *Gnidia glauca* on lipid in HFD-Induced obese rats.

TREATMENT (mg/kgbw)	Lipid Profiles (mmol/L)				
	TG	TC	HDL-C	LDL-C	VLDL
Normal Control	1.44 ± 0.11^b^	1.62 ± 0.13^d^	1.10 ± 0.16^c^	0.50 ± 0.16^b^	0.29 ± 0.02^b^
Negative Control	2.74 ± 0.52^a^	4.34 ± 0.13^a^	0.64 ± 0.21^d^	3.70 ± 0.16^a^	0.54 ± 0.10^a^
Positive Control	1.02 ± 0.16^bc^	1.78 ± 0.18^cd^	1.34 ± 0.11^bc^	0.48 ± 0.13^b^	0.20 ± 0.03^bc^
HFD+200 mg/kg	0.98 ± 0.13^bc^	2.12 ± 0.13^b^	1.36 ± 0.15^bc^	0.52 ± 0.11^b^	0.20 ± 0.03^bc^
HFD+250 mg/kg	0.96 ± 0.11^c^	1.90 ± 0.12^bc^	1.50 ± 0.10^ab^	0.40 ± 0.13^b^	0.19 ± 0.02^c^
HFD+ 300 mg/kg	0.84 ± 0.15^c^	1.86 ± 0.15^cd^	1.76 ± 0.11^a^	0.36 ± 0.15^b^	0.17 ± 0.03^c^

Results are expressed as Means ± SD for five animals per group. The means of various lipid profiles within respective columns followed by a superscript of similar lower-case letter (such as [**a** & **a**b], [**b** & **b**c], [**c**, b**c** & **c**d] or [**d** & c**d**]) are not significantly different from each other (*p* > 0.01). However, in each column, means followed by superscripts of different lower-case letter (such as **a**, **b**, **c** & **d**) are significantly different from each other (*p* ≤ 0.01). Inferential statistics were performed using One way-ANOVA to test for statistical differences among studied groups. Tukey's post hoc test was then done for pairwise separation and comparison of means.

Administration of rats with DCM leaf extract of *G. glauca* caused a significant reduction of the total cholesterol levels than in the negative group rats (*p* ≤ 0.01). Nonetheless, the amount of total cholesterol in rats administered with 300 mg/kg body weight of the extract was not significantly different from that of rats in the normal control group (*p* > 0.01; Table 6). Treatment of rats with extract doses of 200 and 300 mg/kg body weight showed statistically similar levels of total cholesterol as positive control group rats (*p* > 0.01; Table 6).

A dose-dependent elevation of HDL-C levels was observed in rats treated with the three extract doses (Table 6). The results indicated that the levels of HDL-C of rats in the negative control group were significantly low as compared to rats treated with the three extract doses and the reference drug, Orlistat (*p* ≤ 0.01; Table 6). Further, there was no significant difference in levels of HDL-C between rats treated with the reference drug, Orlistat, and rats treated with the extract at the concentrations of 200 and 250 mg/kg body weight (*p* > 0.01; Table 6). On the other hand, HDL-C levels were significantly increased in the group of rats treated with 300 mg/kg body weight of the plant extract (*p* ≤ 0.01; Table 6).

The results demonstrated significantly high levels of LDL-C in the negative control group of rats relative to the extract-treated and Orlistat-treated groups of rats (*p* ≤ 0.01; Table 6). Nevertheless, the difference in the levels of LDL-C of rats treated with the three extract doses were not statistically different from rats treated with the reference drug, Orlistat (*p* > 0.01; Table 6). It is worth noting that the levels of LDL-C of rats in the normal control group and those of rats treated with the plant extract were comparable (*p* > 0.01; Table 6).

The levels of VLDL decreased as the concentration of the extract increased, however, there was no significant difference in VLDL levels among the administered dosages of the plant extract (*p* > 0.01; Table 6). Results indicated that the HFD-induced untreated obese rats had significantly increased levels of VLDL as compared to extract-treated and Orlistat-treated rats (*p* ≤ 0.01; Table 6). Treatment of rats with the three extract doses showed comparable levels of the VLDL with Orlistat-treated rats (*p* > 0.01; Table 6). The extract appeared to normalize levels of VLDL since levels of VLDL of rats in the normal control group were comparable to those of rats treated with the reference drug, Orlistat (*p* > 0.01; Table 6).

### 3.6 Effect of DCM leaf extract of *Gnidia glauca* on body adiposity index (BAI) and atherogenic index (AI) in HFD-Induced obese rats

As depicted in Table 7, administration of DCM leaf extract of *G. glauca* in HFD-induced obese rats caused a significant decrease in the atherogenic index and adiposity index as compared to the HFD-induced untreated obese rats in the negative control group (*p* ≤ 0.01; Table 7). Besides, administration of the extract doses of 250 and 300 mg/kg body weight in rats resulted in a significantly low body adiposity index as compared to Orlistat-treated group of rats (*p* ≤ 0.01; Table 7). Interestingly, the body adiposity index of rats in the extract treated groups were not significantly different from that of rats in the normal control group (*p* > 0.01; Table 7). Results also revealed that the atherogenic indices of rats treated with the three extract doses were significantly lower than those of rats in the normal control group (*p* ≤ 0.01; Table 7). Further, treatment of rats with the extract dose of 300 mg/kg body weight attenuated levels of atherogenic than as observed in Orlistat-treated group of rats (*p* ≤ 0.01; Table 7).

**Table 7:** Effects of DCM leaf extract of *Gnidia glauca* on body adiposity index (Bai) and atherogenic index (AI) in HFD-Induced obese rats.

Treatment	Body Adiposity Index (BAI) (%)	Atherogenic Index (AI) (%)
Normal Control	4.1 ± 0.2^c^	0.1 ± 0.1^b^
Negative Control	6.9 ± 0.2^a^	0.6 ± 0.1^a^
Positive Control	5.1 ± 0.3^b^	-0.1 ± 0.1^c^
HFD+200 mg/kg	4.9 ± 0.3^bc^	-0.1 ± 0.1^c^
HFD+250 mg/kg	4.3 ± 0.1^c^	-0.2 ± 0.1^cd^
HFD+ 300 mg/kg	4.2 ± 0.2^c^	-0.3 ± 0.1^d^

Results are expressed as Means ± SD for five animals per group. The means of Body Adiposity Index (BAI) and Atherogenic Index (AI) within respective columns followed by a superscript of similar lower-case letter (such as [**a**], [**b** & **b**c], [**c**, b**c** & **c**d] or [**d** & c**d**]) are not significantly different from each other (*p* > 0.01). However, in each column, means followed by superscripts of different lower-case letter (such as **a**, **b**, **c** & **d**) are significantly different from each other (*p* ≤ 0.01). Inferential statistics were performed using One way-ANOVA to test for statistical differences among studied groups. Tukey's post hoc test was then done for pairwise separation and comparison of means.

### 3.7 Effects of DCM leaf extract of *Gnidia glauca* on fasting blood glucose levels of HFD-Induced obese laboratory rats

**Fig. 7:**
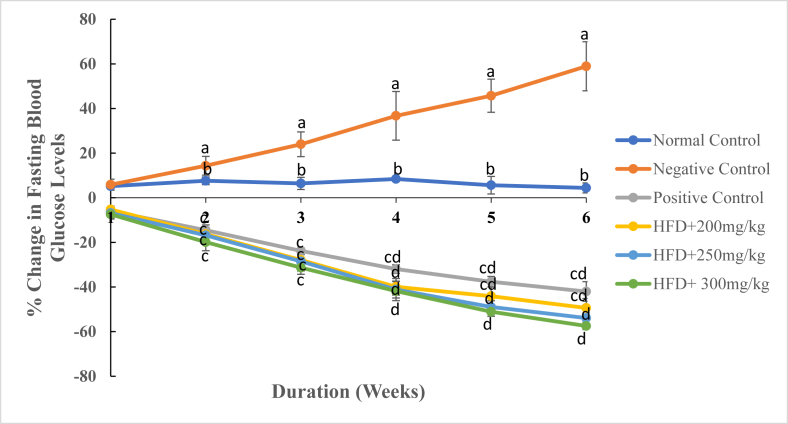
The mean percentage change in fasting blood glucose levels of experimental animals treated with the DCM leaf extract of *Gnidia glauca* for 6 weeks. Each point on the curve represents the mean of the replicate measurement n = 5 expressed as Mean ± S.D. The weekly percentage changes in fasting blood glucose levels among the studied groups with a similar lower-case letter (such as [**c** & **c**d] or [**d** & c**d**) are not significantly different from each other (*p* > 0.01). However, the weekly percentage changes in fasting blood glucose levels with different lower-case letters (such as **a**, **bc** & **d**) indicates statistical difference in means among the studied groups (*p* ≤ 0.01). Inferential statistics were performed using One way-ANOVA to test for statistical differences among studied groups. Tukey's post hoc test was then done for pairwise separation and comparison of means.

**Fig. 8:**
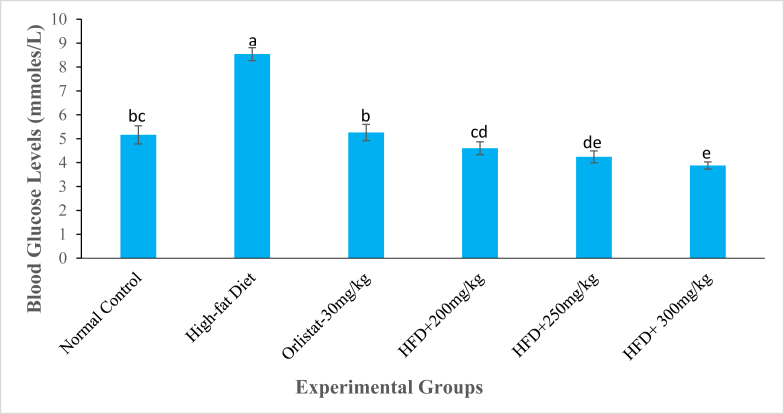
The fasting blood glucose levels (mmole/L) of experimental animals on the 6^th^ week of treatment with the DCM leaf extract of *Gnidia glauca*. Each bar graph represents the mean replicate measurement n = 5 expressed as Mean ± S.D. The bar graphs with a similar lower-case letter (such as [**b** & **b**c], [**c**, b**c** & **c**d] or [e & ed]) among experimental groups are not significantly different from each other (*p* > 0.01). The bar graphs with different lower-case letters (such as **a**, **b**, **cd** & **e**) are statistically different from each other (*p* ≤ 0.01). Inferential statistics were performed using One way-ANOVA to test for statistical differences among studied groups. Tukey's post hoc test was then done for pairwise separation and comparison of means.

As results indicated, treatment with the DCM leaf extract of *G. glauca* and reference drug, Orlistat, caused a reduction in levels of fasting blood glucose in HFD-induced obese rats from the 1^st^ to the 6^th^ week of the study period (Fig. 7). However, rats in the normal control and negative control groups indicated an increase in fasting blood glucose levels throughout the experimental period (Fig. 7). Rat models administered with DCM leaf extract of *G. glauca* at dose levels of 200 and 300 mg/kg body weight caused a decrease in levels of blood glucose from -5.26 ± 1.54% and -7.43 ± 1.67% in the 1^st^ week to -49.37 ± 4.22% and -57.45 ± 1.62% in the 6^th^ week (Fig. 7). On the contrary, rats in the negative control group indicated a percentage increase in fasting blood glucose levels from 5.85 ± 2.47% in the 1^st^ week to 58.93 ± 11.00% in the 6^th^ week of the study period (Fig. 7). On the 42^nd^ day of study, results showed that rats in the negative control group had increased levels of blood glucose than all other experimental groups (Fig. 8). However, the extract-treated rats indicated adose-independent significant decrease in blood glucose levels during the same week (Fig. 8). Besides, the hypoglycemic effects of the extract were higher than those observed in the Orlistat-treated rats in the same period (Fig. 8).

### 3.8 Effects of DCM leaf extract of *Gnidia glauca* on rectal body temperature in HFD induced obese laboratory rats

**Fig. 9:**
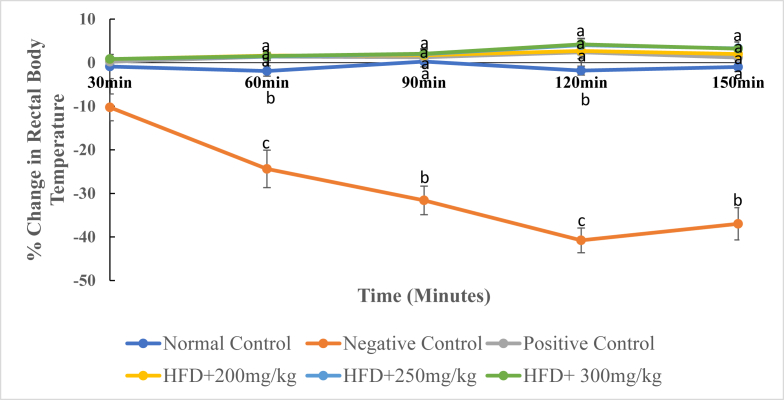
The mean percentage change in rectal body temperature of experimental animals treated with the DCM leaf extract of *Gnidia glauca* for 6 weeks. Each point on the curve represents the mean of the replicate measurement n = 5 expressed as Mean ± S.D. The weekly percentage changes in rectal body temperature among the studied groups with a similar lower-case letter (such as ‘**a**’) are not significantly different from each other (*p* > 0.01). However, the weekly percentage changes in rectal body temperature with different lower-case letters (such as **a**, **b** & **c**) indicates statistical difference in means among the studied groups (*p* ≤ 0.01). Inferential statistics were performed using One way-ANOVA to test for statistical differences among studied groups. Tukey's post hoc test was then done for pairwise separation and comparison of means.

As depicted in Fig. 9, administration of the DCM leaf extract of *G. glauca* and reference drug, Orlistat, caused a gradual increase in the rectal body temperature of rats (Fig. 9). However, rats in the negative control groups indicated a decrease in the rectal body temperature throughout the study period (Fig. 9).

**Fig. 10:**
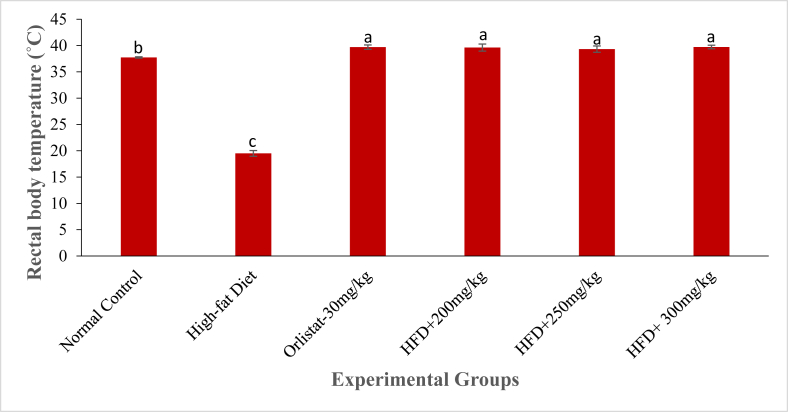
The rectal body temperature (˚C) of experimental animals on the 6^th^ week of treatment with the DCM leaf extract of *Gnidia glauca*. Each bar graph represents the mean replicate measurement n = 5 expressed as Mean ± S.D. The bar graphs with a similar lower-case letter (such as ‘**a**’) among experimental groups are not significantly different from each other (*p* > 0.01). The bar graphs with different lower-case letters (such as **a**, **b** & **c**) are statistically different from each other (*p* ≤ 0.01). Inferential statistics were performed using One way-ANOVA to test for statistical differences among studied groups. Tukey's post hoc test was then done for pairwise separation and comparison of means.

Treatment with the DCM leaf extract of *G. glauca* at dosages of 250 mg/kg and 300 mg/kg body weight caused a percentage increase in rectal body temperature from 0.85 ± 0.45% and 0.84 ± 0.22% in the 30^th^ min to 3.29 ± 1.50% and 3.21 ± 1.52% in the 150^th^ min respectively (Fig. 9). The rats in the negative control group indicated a significant decrease in the rectal body temperature from the 30^th^ to the 150^th^ minute (*p* ≤ 0.01; Fig. 9). For instance, at 30^th^ min, the percentage decrease in rectal body temperature of rats was -10.25 ± 3.07% while at 150^th^ min it was -36.98 ± 3.70% (Fig. 9). Treatment with a standard drug (Orlistat at 30 mg/kg, p.o.) caused a percentage increase in rectal body temperature from 0.21 ± 0.42% in the 30^th^ min to 1.19 ± 1.11% in the 150^th^ min. The rats in the normal control group showed a slight decrease in rectal body temperature from 2.51 ± 1.17% in the 60^th^ to -0.98 ± 1.06% in the 150^th^ minute of study (*p* > 0.01; Fig. 9). As depicted in Fig. 10, the HFD-fed untreated rats showed a highly reduced levels of rectal body temperature on the 150^th^ minute of study. However, on the same time point, the Orlistat-treated rats and extract treated rats indicated relatively high levels rectal body temperature relative to HFD-fed untreated obese rats (*p* < 0.01; Fig. 10).

### 3.9 Effects of DCM leaf extract of *Gnidia glauca* on feed intake in HFD-Induced obese rats

**Fig. 11:**
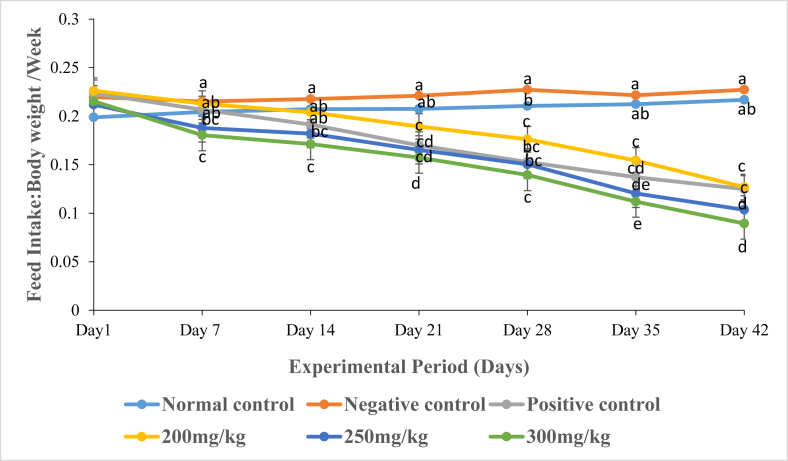
The graph depicts the ratio of feed intake to body weight of rats per week following administration of the DCM leaf extract of *Gnidia glauca* for 6 weeks. Each bar represents the replicate measurement n = 5 expressed as Mean ± S.D at 99% confidence interval. The weekly changes in feed intake among the studied groups with a similar lower-case letter (such as [**a** & **a**b], [**b**, a**b**, & **b**c], [**c**, b**c** & **c**d], [**d**, c**d** & **d**e] or [**e** & d**e**]) are not significantly different from each other (*p* > 0.01). However, the weekly percentage changes in obesity index of rats with different lower-case letters (such as **a**, **b**, **c**, **d** & **e**) indicates statistical difference in means among the studied groups (*p* ≤ 0.01). Inferential statistics were performed using One way-ANOVA to test for statistical differences among studied groups. Tukey's post hoc test was then done for pairwise separation and comparison of means.

Results showed a significant increase in the ratio of the mean cumulative feed intake to body weight of rats in the negative control group as compared to those of rats treated with the three extract doses and the reference drug, Orlistat (*p* ≤ 0.01; Fig. 11). Treatment of rats with the extract at dose of 200 mg/kg body weight caused a decrease in the ratio of total feed intake to body weight of rats from 0.22 in the 1^st^ week to 0.13 in the 6^th^ week of study (Fig. 11). Similarly, treatment of rats with the extract dosage of 300 mg/kg body weight caused a decrease in the ratio of total feed intake to body weight of rats from 0.18 in the 1^st^ week to 0.08 in the 6^th^ week of study (Fig. 11). Treatment of rats with the reference drug, Orlistat, caused a decrease in the ratio of total feed intake to body weight of rats from 0.21 in the 1^st^ week to 0.12 in the 6^th^ week of the study (Fig. 11). However, provision of normal chow diet (rodent pellet and water *ad libitum*) to normal rats in the normal control group progressively the ratio of total feed intake to body weight of rats from 0.20 in the 1^st^ week to 0.22 in the 6^th^ week of the experimental period (Fig. 11).

### 3.10 The Concentration of compounds identified in DCM leaf extract of *Gnidia glauca*

The GC-MS analysis of DCM leaf extract of *G. glauca* revealed the presence of 17 compounds (Table 8). Based on analysis results, γ-Sitosterol (18.8 ± 1.0 mg/kg) had the highest concentration followed by Phenol, 2,4-bis (1,1-dimethylethyl)- (11.2 ± 1.4 mg/kg), Phytol (11.0 ± 1.2 mg/kg), Octadecanoic acid (Stearic Acid) (10.7 ± 1.6 mg/kg), Phytol acetate < E->(10.7 ± 1.3 mg/kg), Gallocatechin–catechin flavan (10.4 ± 1.0 mg/kg), Flavonols (10.2 ± 1.6 mg/kg) and others (Table 8).

**Table 8:** The concentrations of compounds identified in DCM leaf extract of *Gnidia glauca*.

RT	Compound Name	Concentration (mg/kg)
15.12	3,7-dimethyl-2,6-octadiene-1-ol ​acetate (Neryl acetate)	9.8 ± 1.8
18.20	Phenol, 2,4-bis (1,1-dimethylethyl)-	11.2 ± 1.4
21.53	Flavonols	10.2 ± 1.6
24.92	Catechins	9.3 ± 2.1
25.96	Naringenin chalcone	7.7 ± 1.6
26.35	9,12,15-Octadecatrienoic acid, (Z,Z,Z)-(α-Linolenic acid)	9.7 ± 2.9
26.98	Luteolin	9.8 ± 2.6
27.90	Eicosapentaenoic acid	7.6 ± 0.9
28.48	Docosahexaenoic acid	7.9 ± 0.4
30.07	Phytol	11.0 ± 1.18
30.79	γ-Sitosterol	18.8 ± 1.0
32.23	Gallocatechin-catechin flavan	10.4 ± 1.0
34.33	Phytol acetate < E->	10.7 ± 1.3
35.48	Stigmasterol	7.8 ± 2.2
36.29	Squalene	7.5 ± 0.5
36.82	α-Amyrin	5.3 ± 0.8
37.85	β-Amyrin	7.0 ± 2.5

Concentrations of compounds identified in *Gnidia glauca* leaf extract (mg/kg). Results are expressed as Means ± SD for replicate measurement n = 3. RT is the retention time.

## 4 Discussion

The present study tested the effects of the DCM leaf extract of *G. glauca* in an animal model of dietary obesity. The results showed that consumption of calorically dense high-fat diets increased body weight in the HFD-fed untreated group of rats relative to the normal control group throughout the experimental period. These results confirmed the proposition that chronic exposure to high-fat diet results in obesogenic states that is coupled with positive impairment of the energy balance equation [35]. This could be due to the high rate of acylation of saturated fatty acids into triglycerides that is subsequently stored in the adipose tissue hence weight gain [36]. Besides, post-ingestive effects of high fat diets contribute to weight gain through reduction of satiety signals (leptin, cholecystokinin and peptide YY) and attenuated oxidation of fatty acids [37].

It was also observed that there was a cumulative increase in food intake in obese untreated group of rats relative to other experimental groups. Previous studies have reported that eating behavior is modulated by the brain reward systems through mechanisms that involve the homeostatic need to feed as well as the hedonic and cognitive value of ingestion [37]. Overconsumption of high-fat diets has been associated with lesions in the hippocampus that impair rats’ ability to distinguish between the state of hunger and that of satiety [38]. Chronic exposure to HFD promotes positive energy balance that leads to orexigenic responses due to increased activity of appetite-stimulant neuronal group such a neuropeptide Y (NPY), agouti-related protein (AGRP) and the chemical neurotransmitter gamma-aminobutyric acid (GABA) [39]. These neuronal groups potently stimulate food intake, reduce energy expenditure and inhibit the activity of proopiomelanocortin (POMC) and subsequent generation and signaling of melanocyte-stimulating hormone (MSH) [39]. The observed decrease in the quantity of feed intake upon treatment with the plant extract could be attributed to its potential to increase satiety signals that mediate reduction of feed intake, decreases body weight and increases energy expenditure [39]. The presence of gallocatechin-catechin flavan (a form of condensed tannins) in *G. glauca* leaf extract contributes to reduction in feed intake by decreasing palatability [40]. Palatability is reduced because tannins are astringent. Astringency is the sensation caused by the formation of complexes between tannins and salivary glycoproteins. Low palatability depresses feed intake. Reduction of digestibility negatively influences intake because of the fullness effect associated with undigested feedstuff [40]. Besides, condensed tannins influence fat digestion through inhibition of small-intestine micelle formation and the inhibition of α-glucosidase activity leading to a decrease in triacylglycerol absorption [41]. The reduction of absorption of triglycerides would ultimately result in reduced body fat mass [42].

Oral administration of *G. glauca* leaf extract caused a significant reduction in body weight as compared to obese untreated rats (*p* ≤ 0.01). This effect was also comparable to that of the reference drug, Orlistat (*p* > 0.01). Orlistat is a saturated derivative of lipstatin that inhibits the activity of pancreatic lipase resulting in reduced dietary fat absorption [43]. The potential to decrease the body weight of the experimental rats by the extract might be attributed to a singly, additive and/or synergistic effects of the contained phytochemicals. Neryl acetate has been shown to slow gastric emptying and intestinal transit thereby generating an indirect satiety effect [44, 45]. Gallocatechin-catechin flavan reduces the absorption and digestion of triacylglycerol by inhibiting the action of pancreatic lipase [40, 41]. Luteolin was reported to decrease hunger by stimulating the release of satiety signals associated with prolonged gastric emptying [46].

The results also revealed that the obese untreated-rats chronically fed with high-fat diets exhibited persistence increase in obesity index and abdominal circumference from the 1^st^ to 6^th^ week of study. Besides, there was a 2-3-fold increase in fat pad weights (Mesenteric, Retroperitoneal, parametrial, perirenal, subcutaneous) in HFD-induced untreated obese rats relative to extract-treated and normal control rats. These observations suggest that exposures of rats to calorically dense diets facilitated fat accumulation in the abdominal regions due to the high effective energy content of high-fat diets [47]. Previous studies have reported that chronic exposures to a high-fat diet decreases the resting metabolic rate and diet-induced thermogenesis thereby resulting in higher preferential storage of triglycerides in abdominal regions [48]. Dietary obesity, therefore, leads to an increase in number of adipocytes (hyperplasia) and their size (hypertrophy) [49, 50]. Obesity index and abdominal circumference have been established to be the best predictors of intra-abdominal fat thickness in rats and therefore, of central obesity [48]. In humans, parameters such as the abdominal circumference waist, circumference, waist-hip ratio (WHR), and waist to height ratio (WHtR) have been identified to be simple-reliable estimates of body fat content and are among the established criteria for the diagnosis of human metabolic syndromes [29, 48].

Treatment with *G. glauca* leaf extract decreased obesity index and abdominal circumference throughout the experimental period. The observed reduction in these indices might be due to the presence of terpenoids such as squalene, phytol, α-Amyrin, and β-Amyrin which promotes weight reduction through suppressed *de novo* fatty acid synthesis, increases lipid oxidation and reduces food intake [51]. These compounds reduce body fat by acting on adenylate cyclase that converts ATP to cyclic adenosine monophosphate (cAMP). Cyclic adenosine monophosphate (cAMP) promotes lipolysis, increases the body's basal metabolic rate, and increases the use of body fat, and protein degradation and decreases protein synthesis [52]. Therefore, the general reduction in the bioavailability of circulating lipids results in a decrement in the intra-abdominal fat content [53].

The decrease in the relative weights of the total visceral and subcutaneous fat-depot following treatments with the plant extract may be due to inhibitory effect in the formation of new adipocytes from precursor cells (adipocyte differentiation) or decreased adipocyte size due to fat storage (adipocyte hypertrophy) [54]. Visceral fat mass has been shown to be a significant predictor for metabolic disturbances such as cardiovascular diseases, atherosclerosis, dyslipidemia and hypertension [14]. The reduction in body weight, body adiposity index and depletion of body fat stores could account for the extracts’ protective role against obesity-induced metabolic complications. The body adiposity index is used as a measure of adiposity since the degree of fat tends to increase gradually with obesity [55].

It was also observed that the obese untreated rats chronically fed on a high-fat diet showed a marked increase in the organ weights and organo-somatic index of liver, kidney, lungs, heart and brain relative to extract-treated rats. However, the organ weight and organo-somatic index of the spleen was significantly reduced than those of extract-treated rats (*p* ≤ 0.01). Besides, extract-treated rats showed decreased organ weights and organo-somatic index of liver, kidney, lungs, heart and brain relative to obese untreated-rats. The organ weight and organo-somatic index of spleen in extract-treated rats was higher than that of untreated obese rats. Reduction in organ weights and organo-somatic indices could be associated with the observed decreases in body weights of extract-treated rats. Regulation of hunger and appetite signals plays a key role in maintenance of homeostatic balance. Activation of satiety signals such as leptin, melanocyte-stimulating hormone promotes weight reduction through suppressed feed intake [39]. Moreover, facilitated release of peptide YY (PYY), cholecystokinin (CCK), and glucagon-like peptide-1 (GLP-1) slows down gastric emptying and intestinal transit thereby generating indirect satiety effects [44]. The reduction of absorption of triglycerides would ultimately result in reduced body fat mass and hence organ, body weight and organo-somatic index [42].

Results also revealed that administration of *G. glauca* leaf extract decreased levels of TC, TG, LDL and VLDL with a concomitant increase in HDL relative to obese untreated rats. Obesity causes an adverse pattern of plasma lipoproteins [56]. Facilitated visceral adiposity is associated with dyslipidemia which is characterized by elevated TG and reduced HDL-C concentrations [57]. The TGs are involved in the ectopic accumulation of lipid stores in the liver and adipose tissues and are associated with metabolic syndromes. The elevation of plasma TGs in the untreated group of rats supplied with high-fat diets is indicative of increased *de-novo* lipid biosynthesis [58]. Hypertrophied and hyperplastic adipocytes facilitate the synthesis and release of free fatty acids which are transported to the liver where they are re-esterified in hepatocytes to form triglycerides, packaged into VLDL and secreted into the blood circulation [58]. This contributes to lipotoxicity [58].

High dietary intake of simple carbohydrates and fats can also be converted into triacylglycerols in the liver and exported as VLDLs to the adipose tissues [58]. The adipocytes take up these fatty acids and reconvert them back to triacylglycerols for storage in intracellular lipid droplets. The loss of triacylglycerol converts some VLDL to intermediate-density lipoprotein (IDL); further loss of triacylglycerol from VLDL remnants produces low-density lipoprotein (LDL). The LDL is rich in cholesterol and cholesteryl esters and contains apoB-100 as their major apolipoprotein. The LDLs transports cholesterol to extrahepatic tissues that have specific plasma membrane receptors that recognize apoB-100. Intake of foods rich in cholesterol increases levels of LDL since cholesterol enters blood circulation in the form of LDL. The buildup in circulating levels of cholesterol results in hypercholesterolemia which has been associated with plaque formation in the arteries leading to atherosclerosis and stroke [56, 59].

The observed reduction of lipid profiles might be attributed to the effects of the extract's bioactive compounds in augmentation of satiety, suppression of absorption and digestion of dietary lipids as well as inhibition of pancreatic lipase activity [60, 61]. The presence of long-chain polyunsaturated fatty acids such as omega-3 fatty acids (alpha-linolenic acid, docosahexaenoic acid, and eicosapentanoic acid), and omega-9 fatty acid (oleic acid) have been shown to increase the release of satiety hormones such as cholecystokinin (CCK) [62]. The CCK delays gastric emptying and produces a subsequently increased feeling of satiety and a decreased appetite [63]. Phytosterols such as stigmasterol and γ-sitosterol exhibit antihyperlipidemic effect through reduction of serum total cholesterol and triglycerides [64]. Catechins and squalene promote a reduction of body fat by inducing fat oxidation thermogenesis [65, 66].

The contribution of caloric intake to obesity development in this model elicited a significant increase in atherogenic index in HFD-induced untreated obese rats than was observed in rats treated with *G. glauca* leaf extract. Lipid profile and atherogenic index have been shown to be significant predictors for metabolic disturbances including atherosclerosis, cardiovascular diseases, hypertension and dyslipidemia [20]. Any positive change in the levels of lipids make individuals to be more inclined to develop atherosclerotic plaques, cardiovascular diseases and endothelial dysfunction [20].

Findings of the present study also revealed increased levels of blood glucose in HFD-induced untreated obese rats relative to the extract-treated group of rats. Raised levels of circulating blood glucose is characteristic of hyperglycemia as a results of an absolute insulin deficiency and/or insulin resistance [67]. Obesity-related diabetes is mainly associated with insulin resistance and/or hyperinsulinemia due to reduced number of insulin receptors, impaired insulin-receptor binding and disruption in post-receptor insulin signalling transduction [68]. The probable mechanisms for antidiabetic potential of the extract includes restoration of insulin sensitivity, facilitation of uptake of blood glucose by peripheral tissues mediated by an insulin dependent glucose transporter, GLUT-IV, potentiation of insulin release from pancreatic beta cells of Islet of Langerhans as well as elevation of the peripheral glucose utilization [69]. Similarly, *Panax quinquefolius* was shown to confer its hypoglycemic effects through slowing the digestion of food, decrease of the rate of carbohydrate absorption into portal hepatic circulation and increase in insulin release and sensitivity [70]. Chronic exposure to high-fat diet enhances deposition of toxic lipid metabolites (such as fatty acyl CoA, ceramide and diacylglycerol) in pancreatic beta cells, adipocytes, muscle, liver and arterial tissues resulting in insulin resistance, beta-cell dysfunction and accelerated atherosclerosis in type 2 diabetes [71]. Insulin resistance is a fundamental aspect of the etiology of type 2 diabetes and is also linked to a wide array of other pathophysiologic sequelae including hypertension, hyperlipidemia, and atherosclerosis [71].

It was also observed that chronic exposure to high fat diets caused a reduction in thermogenic effect of food as evidenced by decreased rectal temperature in obese untreated rats. Overfeeding with high caloric diet results in a positive energy balance accompanied by a decrease in energy expenditure, which substantially lowers fecal energy loss hence decreased rectal body temperatures as recorded in obese untreated rats [72, 73]. Brown adipose tissue (BAT) is specialized in adaptive thermogenesis through lipid oxidation-mediated heat generation by the uncoupling protein-1 (UCP-1) during the mitochondrial respiratory chain [74]. Activation of adrenergic receptors (ARs) on brown adipose tissue increases lipolysis and fatty acid oxidation resulting in heat production [75]. Chronic exposure to high-fat diet mediate under-expression and unresponsiveness of ARs and a general change in BAT, often, leading to a dysfunction of heat production [76]. As observed, the thermogenic competence of the obese rats might have been increased pharmacologically by administration of the graded doses of DCM leaf extract of *G. glauca*. Similarly, high fat-induced obese rats showed a remarkable reduction in rectal body temperature however, rats treated with *Moringa oleifera* at doses of 200 mg/kg and 400 mg/kg body weight indicated increased the rectal body temperature [24].

Generally, the observed medicinal value of *G. glauca* in the management of obesity lies in the contained phytochemicals whose synergistic effects mediate regulation of various pathways including reduction in lipid absorption, decrease in energy intake, increase in energy expenditure, decrease in pre-adipocyte differentiation and proliferation, decrease in lipogenesis and increase lipolysis as well as free radical scavenging activities [22, 77]. Multiple-phytochemicals combinations may result in synergistic effects that increase their bioavailability and action on multiple molecular targets, thus offering superior therapeutic advantage over treatments with single a chemical compound [5].

## 5 Conclusion

The present study focused on the determination of *in vivo* anti-obesity effects of the DCM leaf extract of *G. glauca* in HFD-induced obese rats. The results indicated that *G. glauca* exhibited its anti-obesity effects through the reduction in body weight, obesity index (OI), abdominal circumference, organ weights, organo-somatic index, total fat content, atherogenic index (AI), body adiposity index (BAI) and feed intake. Besides, a decrease in levels of serum glucose, triglycerides, total cholesterol, LDL-C, VLDL and an increase in HDL-Cs are indicative of the *G. glauca* leaf extract's anti-obesity potential.

The major drawback of the use of polygenic models of study is the lack of reproducibility and inconsistency of generated data. This is due to the variability of micronutrient and macronutrient composition, and in flavor, energy density, palatability, and physical formulation. These, therefore, results in differences in caloric intake, body weight, and body composition. Furthermore, in polygenic diet-induced obesity models, animals are usually restricted to a compulsory type of diet throughout the light–dark cycle, often for periods of many weeks without any choice. This default position is monotonous and poorly depicts the meal pattern and feeding habits of human populations. Therefore, there's a need to develop polygenic models that better mimic human behaviors with greater emphasis on dietary choices, feeding and drinking regimen, binge-eating, and further investigations on motivational and compulsion to overeat.

The observed anti-obesity effects in extract-treated rats might be attributed to a single, additive and/or synergistic effect of the contained phytochemicals. These phytocompounds contributes in the reduction in lipid absorption, decreases energy intake, increases energy expenditure and regulates differentiation and proliferation of pre-adipocytes. The findings of the present study, therefore, affirm the folkloric use of this plant as an alternative therapy for the management of obesity and associated complications such as type 2 diabetes mellitus. The generated data provide ‘qualified leads’ in the synthesis of a new and effective anti-obesity drug from *G. glauca*. In an attempt to conform with guidelines of the National Centre for the Replacement, Refinement, and Reduction of Animals in Research, we de-epithelialized key tissues and have established a cell-culture system in our laboratory. The cells from these tissues will form a basis for further research on how *G. glauca* mediate expression of certain factors responsible for obesity-induced oxidative damage. Further studies on the toxicological effect of *G. glauca* will help decipher its safety profiles *in vitro*. The cell-culture system will go a long way in the reduction in animal use in an attempt to generate positive evidence to support this hypothesis.

## Declarations

### Author contribution Statement

Wycliffe Makori Arika: Performed the experiments; Analyzed and interpreted the data; Contributed reagents, materials, analysis tools or data; Wrote the paper.

Cromwell Mwiti Kibiti, Joan Murugi Njagi: Analyzed and interpreted the data; Contributed reagents, materials, analysis tools or data.

Mathew Piero Ngugi: Conceived and designed the experiments; Analyzed and interpreted the data; Contributed reagents, materials, analysis tools or data.

### Funding Statement

This research did not receive any specific grant from funding agencies in the public, commercial, or not-for-profit sectors.

### Competing interest Statement

The authors declare no conflict of interest.

### Additional information

No additional information is available for this paper.
